# Postoperative chylous ascites after total gastrectomy successfully treated using peritoneovenous (Denver) shunt: a case report

**DOI:** 10.1186/s40792-022-01447-0

**Published:** 2022-05-10

**Authors:** Shinya Sakamoto, Nobuo Takata, Yoshihiro Noda, Kazuhide Ozaki, Takehiro Okabayashi

**Affiliations:** 1 Department of Gastroenterological Surgery, Kochi Health Science Center, 2125-1 Ike, Kochi, 781-8555 Japan; 2 Department of Radiology, Kochi Health Science Center, 2125-1 Ike, Kochi, 781-8555 Japan

**Keywords:** Gastric cancer, Postoperative chylous ascites, Para-aortic lymph node dissection, Peritoneovenous shunt

## Abstract

**Background:**

Chylous ascites (CA) is a rare complication of gastrectomy for gastric cancer. While most cases of postoperative CA improve with medication or nutritional support, some are refractory to conservative treatment. A peritoneovenous shunt (PVS) may help patients who are poor candidates for surgery. However, PVS placement for postoperative CA after gastroenterological surgery has been rarely reported. Herein, we present a case of postoperative CA following total gastrectomy with para-aortic lymphadenectomy, treated successfully by PVS placement.

**Case presentation:**

A 74-year-old man who underwent total gastrectomy with para-aortic lymph node dissection was hospitalised because of insufficient oral intake and dehydration. His abdomen was markedly distended with severe bilateral lower extremity oedema. On admission, abdominal computed tomography (CT) showed a high volume of ascites and no signs of cancer recurrence. Accordingly, postoperative CA resulting from drainage of fluid on paracentesis was diagnosed. Despite nutritional support, diuretics, and octreotide administration, his abdominal distension and nutritional status did not improve. We could not identify the sites of lymphatic leakage in the three intranodal lymphangiographies followed by CT. Although we considered a surgical treatment in our patient, we decided against it. Because we could not identify lymphatic leakage site during lymphangiography, surgical treatment might have a potential failure of detection and closure of leakage site. Furthermore, the patient’s general condition was poor because of malnutrition resulting from the loss of lymphatic fluid. Consequently, we decided to place PVS. After PVS placement, his abdominal distension improved rapidly, and he was discharged without serious complications. Thirteen months after PVS, patient has no relapse of abdominal distention and nutrition status has improved.

**Conclusion:**

PVS might be a good option to manage refractory postoperative CA, as the leakage point cannot be detected on lymphangiography.

## Background

Chylous ascites (CA) occurs rarely after gastrectomy for gastric cancers [1]. However, clinicians should be aware that this complication may occur in patients who undergo para-aortic lymph node dissection (PALN). The incidence of CA was reportedly 3.8–11.8% among those who underwent gastrectomy with D2 plus PALN [2, 3]. Most cases of postoperative CA improve with conservative treatment. Surgical treatment is considered in cases refractory to conservative treatment. A peritoneovenous shunt (PVS) is considered an option for patients who are poor candidates for surgery [4]. However, PVS placement for postoperative CA after gastroenterological surgery has been rarely reported. Herein, we present a case of postoperative CA following total gastrectomy with PALN, treated successfully by PVS placement.

## Case presentation

**Fig.1:**
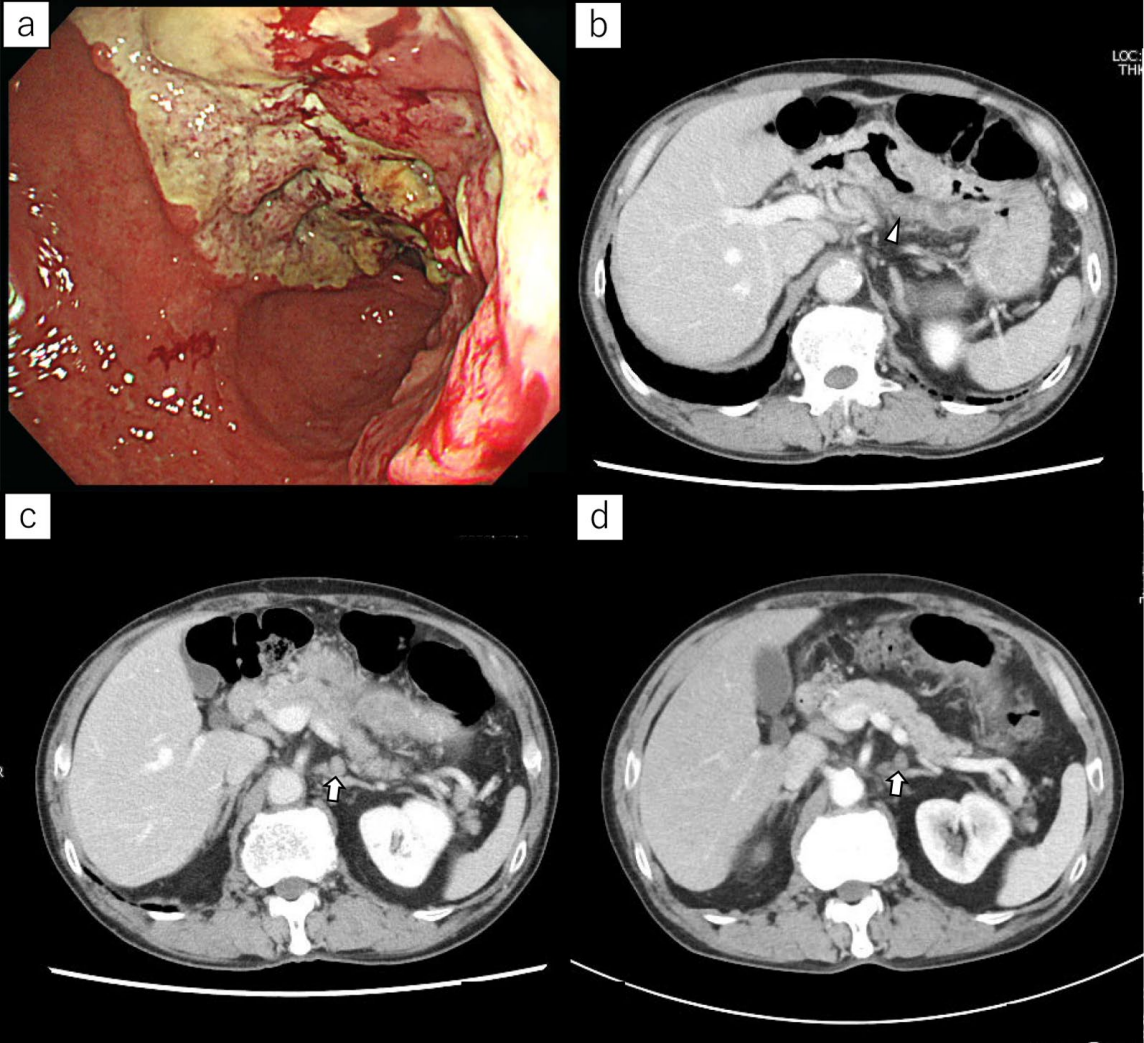
**a** Esophagogastroduodenoscopy revealed huge ulcerative lesion on the lesser curvature of stomach. **b** Abdominal CT revealed wall thickness of stomach (arrowhead) and enlarged lymph node along the left gastric artery and its branches. **c** Abdominal CT revealed enlarged para-aortic lymph node (arrow). **d** Para-aortic lymph node was slightly shrunk after chemotherapy (arrow)

A 74-year-old man was referred to our hospital and was diagnosed with gastric cancer with para-aortic lymph node metastasis (Fig. 1a–c). We introduced chemotherapy and no newly metastatic lesion was appeared. Curative surgery was proposed, but he declined it. The primary lesion was remarkably shrinking but remaining, and he continued to receive chemotherapy. After the patient ﻿received 17 cycles of chemotherapy (S-1 and oxaliplatin), he was admitted to our hospital because of Mallory–Weiss syndrome when he underwent follow-up endoscopic examination. The lesion, which was remarkably shrunk while chemotherapy was introduced, had endoscopically grown. Regular follow-up computed tomography (CT) showed the primary lesion shrank once but gradually increased in size, and the regional and para-aortic lymph nodes slightly shrank (Fig. 1d), and no significant change was observed in diameter after regrowth of the primary lesion. No newly developed metastatic lesions were observed. Gastrectomy and extended lymph node dissection were planned for curative surgery again. He had no history of viral hepatitis, and he did not have drinking habit. Laboratory examination revealed deterioration of liver function after receiving chemotherapy. His serum total bilirubin level was 1.9 mg/dL, a significant increase compared with the previous value of 0.6 g/dL. However, preoperative CT scan did not reveal change suspecting liver cirrhosis including ascites. The patient underwent open total gastrectomy and D2 plus PALN. The patient was allowed oral food intake on postoperative day four. The drain was removed on postoperative day seven. Serous fluid (864 mL) was drained a day before removing the drain. His postoperative course was uneventful, and he was discharged on postoperative day 14. Histological tumour findings were gastric cancer, LM, Post-Less-Ant, ypType 3, 72 × 42 mm, tub1 > pap > tub2, ly1b, v1a, pT1b (SM2), pN0 (0/53), M0, ypStage IA, Grade 1b), according to the Japanese Classification of Gastric Cancer, 15th edition [5].

**Fig.2:**
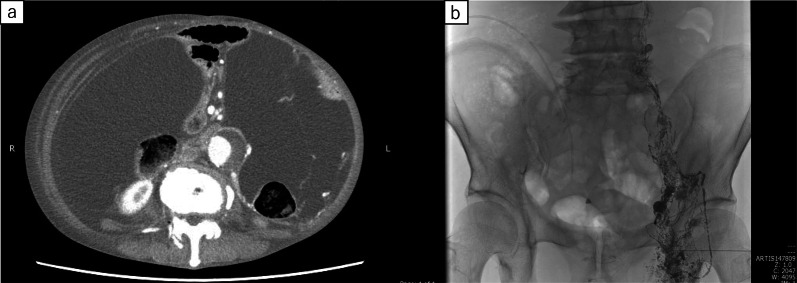
**a** Abdominal CT showed a large volume of ascites and no sign of cancer recurrence. **b** Fluoroscopic image showing injection of Lipiodol through the left inguinal lymph nodes (arrow)

After discharge, his oral intake was not sufficient. One month after surgery, he presented with bilateral lower extremity oedema and hypoalbuminaemia. Three months after gastrectomy, he was readmitted to our hospital because of insufficient oral intake and dehydration. His abdomen was markedly distended, and severe bilateral lower extremity oedema was observed. On admission, abdominal CT showed a high volume of ascites and no cancer recurrence (Fig. 2a). A drainage catheter placed in the peritoneal cavity drained 7,500 mL of white milky fluid with a high triglyceride level (348 mg/dL). Cytological examination revealed no malignancy. The bacterial culture was negative. The patient was diagnosed as having postoperative CA. We could not identify the sites of lymphatic leakage in the three times of intranodal lymphangiography followed by CT (Fig. 2b). He ingested 250–500 kcal fat restriction food orally and received 1020 kcal parenteral nutrition with intralipid supplementation. Abdominal distension and nutritional status did not improve despite starting a fat-restricted diet, parenteral nutrition support, diuretics, and octreotide (300 μg/day) administration. Almost 1500 mL fluid was drained a day when we performed continuous drainage of his ascites. We performed CART [6] for four times almost each 2 weeks, and approximately 8 L of ascites was drained each time. However, his abdominal distention temporally improved, but he complained of abdominal discomfort in a few days after CART. After one week when he underwent CART, his abdomen was significantly distended. Furthermore, his serum albumin levels progressively decreased for 4 weeks after admission. The postoperative CA was refractory to conservative treatment and lymphangiography. Thus, surgical treatment was considered. However, we suspected surgical approach might not be effective because the several times of lymphangiography could not detect the leakage point. Moreover, his general condition was poor due to loss of lymphatic fluid. After the surgical indications were discussed in detail and the patient was given sufficient informed consent before treatment, we decided to place a PVS.

**Fig.3:**
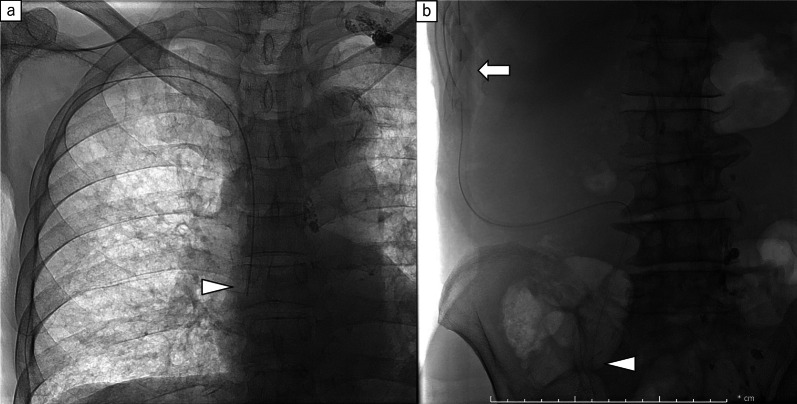
**a** The venous limb of the peritoneovenous shunt was placed with the tip of the catheter located in the lower end of the superior vena cava (arrowhead). **b** The peritoneal limb of the shunt was placed at the tip of the catheter within the pelvic region (arrowhead). The pump was placed in front of the right lower rib cage (arrow)

**Fig.4:**
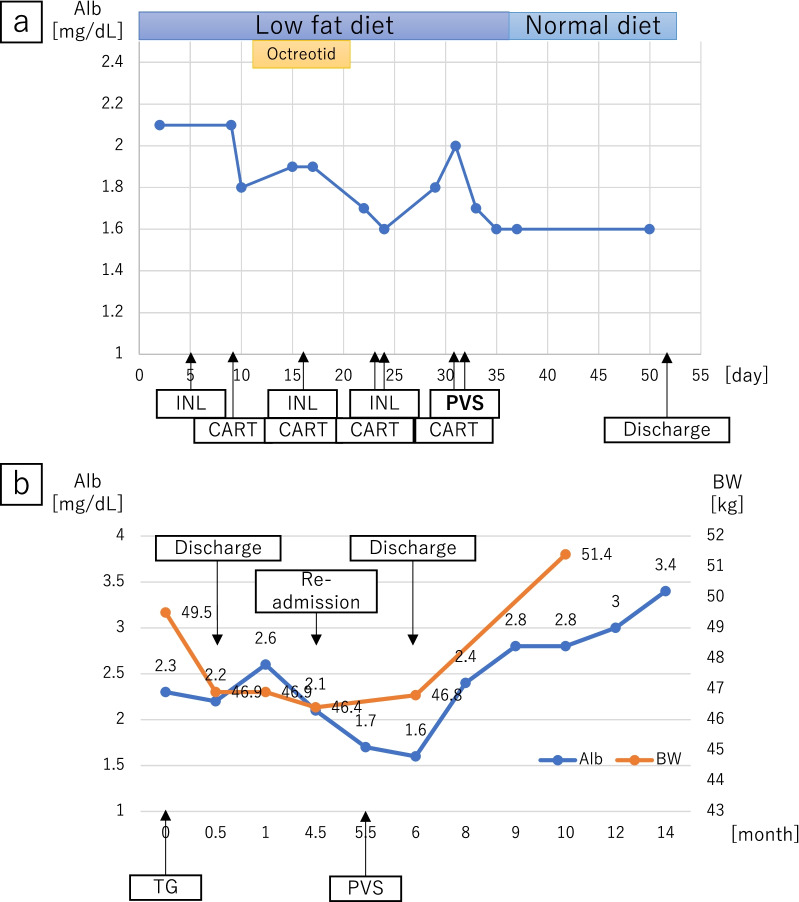
Clinical course. **a** Changes in serum albumin value (Alb) during the second hospitalisation. **b** Changes in serum albumin value (Alb) and body weight (BW) after initial gastrectomy. *CART* cell-free and concentrated ascites reinfusion therapy, *INL* intranodal lymphangiography, *PVS* peritoneovenous shunt, *TG* total gastrectomy

**Fig.5:**
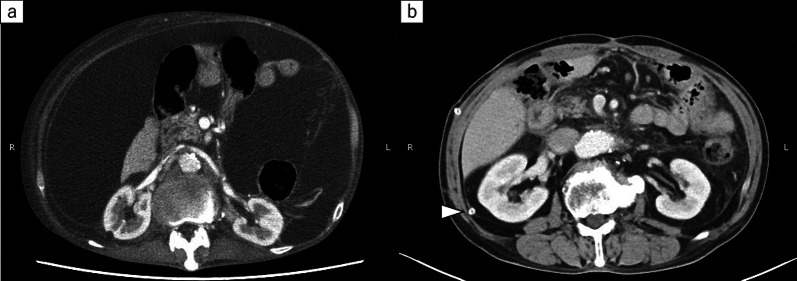
**a** Abdominal CT showed a large volume of ascites before peritoneovenous shunt. **b** Abdominal CT showed no ascites at a follow-up examination in the outpatient clinic. Arrow; the peritoneal limb of the PVS

The peritoneovenous (Denver) shunt was placed on the 31st day. The first incision was made on a firm, non-compressible rib in the right lower rib cage. A pocket was carved to place the pump as planned. The peritoneal limb of the shunt was inserted into the abdominal cavity through an incision. The venous limb of the shunt was inserted from the subclavian vein into the superior vena cava. A second incision was made on the lateral side of the venous access site. The pump was pushed into the pocket, and the venous limb of the shunt was tunnelled under the skin through the superior aspect of the first incision (Fig. 3). The patient pushed the shunt valve ten times twice a day after PVS placement. His abdominal distension drastically improved after placing the PVS. Although laboratory ﻿investigation showed decrease in platelet count (from 10.5 to 4.8 × 10^4^ μL) and in prothrombin time (from 59.5 to 43.5%) in a week, he did not suffer from ﻿severe disseminated intravascular coagulation (DIC) and no serious complications were observed. The patient was discharged on the 51st day (20 days after PVS placement) (Fig. 4a). A follow-up examination in the outpatient clinic at 13 months after PVS placement, he remained asymptomatic without recurrence of ascites and cancer (Fig. 5); nutritional status also showed improvement (Fig. 4b).

## Discussion

Postoperative CA is commonly caused by intraoperative injury to the lymphatic system with subsequent lymphatic leakage into the peritoneal cavity [7]. CA might also occur due to increased pressure in the lymphatics compared to the abdominal cavity and tissue pressure due to adhesions or extrinsic compression of lymphatic vessels [8]. The lymphatic system is an important route for the passage of lipids and water from the intestinal lumen to the vascular system. Loss of lymphatic fluid in the peritoneal cavity can deplete lymphatic fluid, resulting in malnutrition, electrolyte imbalance, and immunosuppression, increasing postoperative mortality [7].

Postoperative CA diagnosis is based on its appearance, a milky and non-purulent fluid in the drainage tube or on paracentesis. Laboratory findings for CA include chylomicron-rich drainage fluid with a triglyceride concentration greater than 110–200 mg/dL [7]. Although lymphangiography reportedly helps identify the site of leakage effectively [9, 10], the leakage point cannot be detected in some cases during lymphangiography owing to a minimal injury to the lymphatics [11]. Yokokawa et al. proposed that lymph flow stasis may cause postoperative CA [12]. CA caused by central lymphatic system obstruction cannot be identified as a leakage site during lymphangiography. In the present case, we could not detect the point of chyle leakage during intranodal lymphangiography. We speculated we could not visually detected leakage point during intra-nodal lymphangiography because the injury point was in the route from intestinal lymphatics or hepatic lymphatics to cisterna chyli.

Conservative therapy is the first line of treatment for CA. And most cases of postoperative CA are treated conservatively [7]. Nutritional support, including low-fat and high-protein diets with medium-chain triglycerides or total parenteral nutrition, and octreotide treatment, decreases lymphatic flow and reduces intestinal fat absorption [13]. Lymphangiography with or without embolisation is also an effective treatment for postoperative CA [9]. Surgical treatment is considered for patients with CA refractory to medical treatment who have also experienced metabolic complications [8, 14]. A laparotomy may be performed for fistula closure. Upon identifying the leakage, suture ligation of the lymphatic vessels may help resolve the leak. PVS is considered an option for patients who are poor candidates for surgery [15]. Although we considered a surgical treatment in our patient, we decided against it. Because we could not identify lymphatic leakage site during lymphangiography, surgical treatment might have a potential failure of detection and closure of leakage site. Furthermore, the patient’s general condition was poor because of malnutrition resulting from the loss of lymphatic fluid. Consequently, we decided to place PVS.

PVS is primarily accepted as a treatment for refractory cirrhotic or malignant ascites [15, 16], and is also indicated in CA [15]. PVS can provide immediate relief from symptoms. However, clinicians should be aware of several severe complications, including DIC, pulmonary oedema, and pulmonary thromboembolism [17]. PVS is generally used as a palliative treatment for patients with severe liver cirrhosis and advanced malignant tumours with relatively short life expectancy. Therefore, the long-term outcomes of this modality remain unclear [18, 19]. Consequently, indication of PVS for cases with expected long-term prognosis should be carefully discussed. Yarmohammadi et al. reported that postoperative CA was permanently resolved after PVS placement in patients whose ascites had resulted from retroperitoneal lymph node dissection for urological malignancy [20]. PVS placement for postoperative CA following a surgical procedure may potentially resolve the complication permanently. In the present case, the patient’s symptoms disappeared, and his nutritional status improved drastically following PVS placement. A follow-up examination in the outpatient clinic, his CA seemed to be healed because CT revealed no ascites. We speculated that improvement of nutrition status and reduction the space around lymphatic fistula because of continuous drainage might cause healing postoperative CA in the patient. The patient experienced no serious complications despite malnutrition. The postoperative course was generally favourable. We do not plan to remove the PVS because it is difficult to evaluate if PVS is working well.

## Conclusion

The present report describes a case of postoperative CA after total gastrectomy with D2 plus para-aortic lymph node dissection, successfully treated using PVS. PVS might be a good option for poor candidates of surgical treatment to manage refractory postoperative CA, which cannot be detected by lymphangiography.

## Data Availability

All data generated or analysed during this study are included in the published article.

## Abbreviations

CA: Chylous ascites
PALN: Para-aortic lymph node dissection
PVS: Peritoneovenous shunt
CT: Computed tomography
DIC: ﻿Disseminated intravascular coagulation

## References

[1] Hu Y, Huang C, Sun Y, Su X, Cao H, Hu J (2016). Morbidity and mortality of laparoscopic versus open D2 distal gastrectomy for advanced gastric cancer: a randomized controlled trial. J Clin Oncol.

[2] Sano T, Sasako M, Yamamoto S, Nashimoto A, Kurita A, Hiratsuka M (2004). Gastric cancer surgery: morbidity and mortality results from a prospective randomized controlled trial comparing D2 and extended para-aortic lymphadenectomy–Japan Clinical Oncology Group study 9501. J Clin Oncol.

[3] Yol S, Bostanci EB, Ozogul Y, Ulas M, Akoglu M (2005). A rare complication of D3 dissection for gastric carcinoma: chyloperitoneum. Gastric Cancer.

[4] Bhardwaj R, Vaziri H, Gautam A, Ballesteros E, Karimeddini D, Wu GY (2018). Chylous ascites: a review of pathogenesis, diagnosis and treatment. J Clin Transl Hepatol.

[5] Association JGC (2021). Japanese gastric cancer treatment guidelines 2018. Gastric Cancer.

[6] Matsusaki K, Orihashi K (2020). Feasibility, efficacy, and safety of cell-free and concentrated ascites reinfusion therapy (KM-CART) for malignant ascites. Artif Organs.

[7] Weniger M, D'Haese JG, Angele MK, Kleespies A, Werner J, Hartwig W (2016). Treatment options for chylous ascites after major abdominal surgery: a systematic review. Am J Surg.

[8] Ilhan E, Demir U, Alemdar A, Ureyen O, Eryavuz Y, Mihmanli M (2016). Management of high-output chylous ascites after D2-lymphadenectomy in patients with gastric cancer: a multi-center study. J Gastrointest Oncol.

[9] Kim PH, Tsauo J, Shin JH (2020). Lymphangiography with or without embolization for the treatment of postoperative chylous ascites. Ann Vasc Surg.

[10] Kawasaki R, Sugimoto K, Fujii M, Miyamoto N, Okada T, Yamaguchi M (2013). Therapeutic effectiveness of diagnostic lymphangiography for refractory postoperative chylothorax and chylous ascites: correlation with radiologic findings and preceding medical treatment. AJR Am J Roentgenol.

[11] Matsumoto T, Yamagami T, Kato T, Hirota T, Yoshimatsu MT (2009). The effectiveness of lymphangiography as a treatment method for various chyle leakages. Br J Radiol.

[12] Yokokawa H, Katsube T, Miyazawa M, Nishiguchi R, Asaka S, Yamaguchi K (2021). First successful case of percutaneous transabdominal thoracic duct embolization (PTTDE) for chylous ascites resulting from laparoscopic gastric cancer surgery. Int Cancer Conf J.

[13] Lizaola B, Bonder A, Trivedi HD, Tapper EB, Cardenas A (2017). Review article: the diagnostic approach and current management of chylous ascites. Aliment Pharmacol Ther.

[14] Lv S, Wang Q, Zhao W, Han L, Wang Q, Batchu N (2017). A review of the postoperative lymphatic leakage. Oncotarget.

[15] Will V, Rodrigues SG, Berzigotti A (2022). Current treatment options of refractory ascites in liver cirrhosis—a systematic review and meta-analysis. Dig Liver Dis.

[16] Becker G, Galandi D, Blum HE (2006). Malignant ascites: systematic review and guideline for treatment. Eur J Cancer.

[17] Yarmohammadi H, Getrajdman GI (2017). Symptomatic fluid drainage: peritoneovenous shunt placement. Semin Intervent Radiol.

[18] Piccirillo M, Rinaldi L, Leongito M, Amore A, Crispo A, Granata V (2017). Percutaneous implant of Denver peritoneovenous shunt for treatment of refractory ascites: a single center retrospective study. Eur Rev Med Pharmacol Sci.

[19] Tamagawa H, Aoyama T, Inoue H, Fujikawa H, Sawazaki S, Numata M (2020). Therapeutic results of Denver percutaneous peritoneovenous shunt in cancer patients with malignant ascites. J Cancer Res Ther.

[20] Yarmohammadi H, Brody LA, Erinjeri JP, Covey AM, Boas FE, Ziv E (2016). Therapeutic application of percutaneous peritoneovenous (Denver) shunt in treating chylous ascites in cancer patients. J Vasc Interv Radiol.

